# Diagnosis and Treatment of Diseases of the Rectum, Anus, and Contiguous Textures

**Published:** 1896-12

**Authors:** 


### BOOK NOTICE.

Diagnosis and Treatment of Diseases of the Rectum, Anus, and Contiguous Textures. Designed for Practitioners and Students. By S. G. Gant, M. D., Professor of Diseases of the Rectum and Anus, University and Woman's Medical Colleges; Lecturer on Intestinal Diseases in the Scarritt Training-School for Nurses; with two chapters on "Cancer" and "Colotomy" by Herbert William Allingham, F. R. C. S. Eng., Surgeon to the Great Northern Hospital. One volume, royal octavo, 400 pages. Illustrated with 16 full-page chromo-lithographic plates and 115 wood engravings in the text. Extra cloth, $3.50 net; Half-Russia, gilt top, $4.50 net. The F. A. Davis Co., Publishers, 1914 and 1916 Cherry Street, Philadelphia; 117 W. Forty-Second Street, New York; 9 Lakeside Building, Chicago.

This remarkable and most useful book is one of the most elegantly illustrated pieces of book-making it has been our lot to meet with in a long time. It contains sixteen full-page chromo-lithographs, the colors being true to life, and its wood engravings are highly illustrative of the text.

It is divided into thirty-two chapters and each chapter is complete in itself. The style is vividly clear and completely explanatory, so that there need be no excuse for not getting a perfect understanding of the subjects treated. Yet we cannot say that any superfluous language is used. Certain it is that a perusal of any chapter will be found to leave upon the mind a most vivid and positive impression.

The subjects are all classified so well that the reader may obtain what he wants without reading a lot of matter not immediately bearing on the point which he wishes made clear to him. The better to obtain this result the chapters are divided into paragraphs, each of which is so labeled as to show its contents. Still better to reduce the difficulty of finding the subject wanted there is an index by chapters, an index of illustrations, and an alphabetical index. Moreover, the tops of all the right-hand pages are labeled with the contents of the pages.

The illustrations are beautiful in drawing and in color; they are all new, prepared expressly for this work, so that, taking it as a whole, we may say of it that it is a beautiful book.

Those who wish to do rectal work, to make of it a specialty, cannot go on in their career with a knowledge of the latest procedures without a copy of this work.

The first ten pages contain a complete statement of the anatomy of the rectum and anus, with colored plates representing dissections. The author claims, in speaking of the rectum, that the rectum is always empty until just before defecation. He also thinks that the sensation which immediately precedes defecation, is not due either to contact of stool nor to irritants contained in the feces, but that "it is of an organic nature, as a result of some intestinal change which takes place before the mass reaches the rectum." Prolapse of the rectum is well treated and a startling illustration of complete procidentia in colors is given at page 44.

In all medical works that are up to date a great deal of attention is paid to the comparatively new factor in pathology—auto-intoxication. The reader may remember a work on auto-intoxication, published by the F. A. Davis Co., which was reviewed in this journal October, 1894, page 333.

The book now under review shows its advanced stand by devoting an exceedingly interesting chapter to auto-intoxication from the contents of the intestinal canal. It is not possible within the limits of this review to discuss the salient points of this chapter and must refer the reader to the book itself.

In the chapter upon the performing of operations for artificial anus, too much praise cannot be given the author for the minuteness and clearness of his description of the various steps in this important procedure.

A short chapter is given to the subject of foreign bodies in the rectum. An astonishing case is recorded of a man who had accidentally forced into his rectum a knotted stick one inch in diameter and ten inches long. It was removed with the greatest difficulty owing to a natural hook upon the inserted end, produced by cutting off a branch that originally grew there.

A remarkable and novel chapter is that entitled "Railroading as an etiological factor in rectal diseases." This subject has not to the knowledge of the reviewer ever been brought to the attention of the profession before.

According to the author seventy-five per cent. of the men engaged in railroad work have diseases of the rectum directly induced by their occupation. He mentions three different modes in which this effect is brought about: first, irregularity in living; secondly, erect position for long periods of time, and third, the jolting motion of cars and engines. These causes produce a variety of troubles all of which are analyzed and explained in the most satisfactory way.

